# A Comprehensive Review: Mesoporous Silica Nanoparticles Greatly Improve Pharmacological Effectiveness of Phytoconstituent in Plant Extracts

**DOI:** 10.3390/ph17121684

**Published:** 2024-12-13

**Authors:** Diah Lia Aulifa, Bunga Amarilis, Luthfia Nur Ichsani, Devita Salsa Maharani, Ayunda Myela Shabrina, Hanifah Hanifah, Rizky Prasiska Wulandari, Agus Rusdin, Laila Subra, Arif Budiman

**Affiliations:** 1 Department of Pharmaceutical Analysis and Medicinal Chemistry, Faculty of Pharmacy, Universitas Padjadjaran, Jl. Raya Bandung-Sumedang Km. 21, Bandung 45363, Indonesia; rizky21006@mail.unpad.ac.id (R.P.W.); agusrusdin@gmail.com (A.R.); 2 Department of Pharmaceutics and Pharmaceutical Technology, Faculty of Pharmacy, Universitas Padjadjaran, Jl. Raya Bandung-Sumedang Km. 21, Bandung 45363, Indonesia; bunga21004@mail.unpad.ac.id (B.A.); luthfia21001@mail.unpad.ac.id (L.N.I.); devita21001@mail.unpad.ac.id (D.S.M.); ayunda21001@mail.unpad.ac.id (A.M.S.); hanifah21006@mail.unpad.ac.id (H.H.); 3 Department of Pharmacy, Faculty of Bioeconomic, Food and Health Sciences, Universiti Geomatika Malaysia, Kuala Lumpur 54200, Malaysia; laila@geomatika.edu.my

**Keywords:** medicinal plant extracts, mesoporous silica nanoparticles, drug nanocarriers

## Abstract

Medicinal plants are increasingly being explored due to their possible pharmacological properties and minimal adverse effects. However, low bioavailability and stability often limit efficacy, necessitating high oral doses to achieve therapeutic levels in the bloodstream. Mesoporous silica nanoparticles (MSNs) offer a potential solution to these limitations. Due to their large surface area, substantial pore volume, and ability to precisely control pore size. MSNs are also capable of efficiently incorporating a wide range of therapeutic substances, including herbal plant extracts, leading to potential use for drug containment and delivery systems. Therefore, this review aimed to discuss and summarize the successful developments of herbal plant extracts loaded into MSN, focusing on preparation, characterization, and the impact on efficacy. Data were collected from publications on Scopus, PubMed, and Google Scholar databases using the precise keywords “mesoporous silica nanoparticle” and “herbal extract”. The results showed that improved phytoconstituent bioavailability, modified release profiles, increased stability, reduced dose and toxicity are the primary benefits of this method. This review offers insights on the significance of integrating MSNs into therapeutic formulations to improve pharmacological characteristics and effectiveness of medicinal plant extracts. Future prospects show favorable potential for therapeutic applications using MSNs combined with herbal medicines for clinical therapy.

## 1. Introduction

Secondary metabolites derived from plants are the main source of bioactive compounds, which have pharmacological or toxicological effects on living organisms. Biomedical and natural product studies recognize the essential compounds found in herbal medications, such as phenolics, terpenoids, alkaloids, and glycosides, for their therapeutic properties. The ability to neutralize harmful molecules and a wide range of biological functions underscore the significance of the compounds highlight their significance in the treatment and prevention of diseases [1,2].

Medicinal plants have attracted attention due to their potent compounds, which show significantly fewer side effects compared to synthetic medications [3]. However, the presence of obstacles such as limited bioavailability and brief half-lives requires the implementation of techniques to improve stability and effectiveness. Previous studies have investigated various encapsulation methods using matrices such as chitosan [4], alginate [5], liposomes [6], and MSNs [7,8] to address these obstacles.

The use of nanoparticles as drug carriers is a promising method for the targeted delivery of substances at the cellular level [9,10]. These carriers enhance the solubility of poorly absorbed drugs, leading to improved bioavailability, and are also effectively used in drug delivery systems [10]. Another advantage is enhancing the performance of bioactive agents by improving their in vitro solubility and bioavailability. The carriers also improve in vivo stability while inhibiting nonspecific interactions with other molecules [11].

MSN systems could enhance therapeutic effects on specific targets and reduce toxicity. They are also beneficial in ensuring the controlled release of drugs [12,13,14,15]. Their large pore capacity and surface area enable effective loading and release of both hydrophilic and hydrophobic drugs [16,17]. Due to their physicochemical stability, biocompatibility, and clarity of functionalization, MSNs are highly adaptable for therapeutic purposes such as targeted delivery and controlled release [18,19,20,21,22,23,24,25,26].

Considerable interest has been drawn to the incorporation of MSNs with herbal plant extracts [27,28,29]. The ability to encapsulate and protect bioactive substances derived from plant extracts, as well as bioavailability, offers a novel approach in the field of drug delivery systems [30,31,32]. Although various studies have examined the use of MSNs for drug delivery, there is no detailed explanation or deep analysis of how the mechanism can improve herbal plant extract performance in terms of dissolution and pharmacological properties [33,34]. A previous study reported MSNs’ capability to improve natural-derived materials performance. However, the study primarily focused on a single chemical compound and only evaluated its potency in a specific context, particularly cancer [32].

To address the gap, this review aimed to assemble present knowledge on the use of MSNs for improved efficacy of herbal plant extracts. It will provide broad insights into optimizing MSN formulations for therapeutic applications by outlining the successes and challenges in this field. The results are expected to serve as a cornerstone in guiding future study efforts toward the effective use of MSNs with plant extracts, potentially paving the way for innovative therapeutic strategies in combating various diseases.

## 2. Methodology

**Figure 1:**
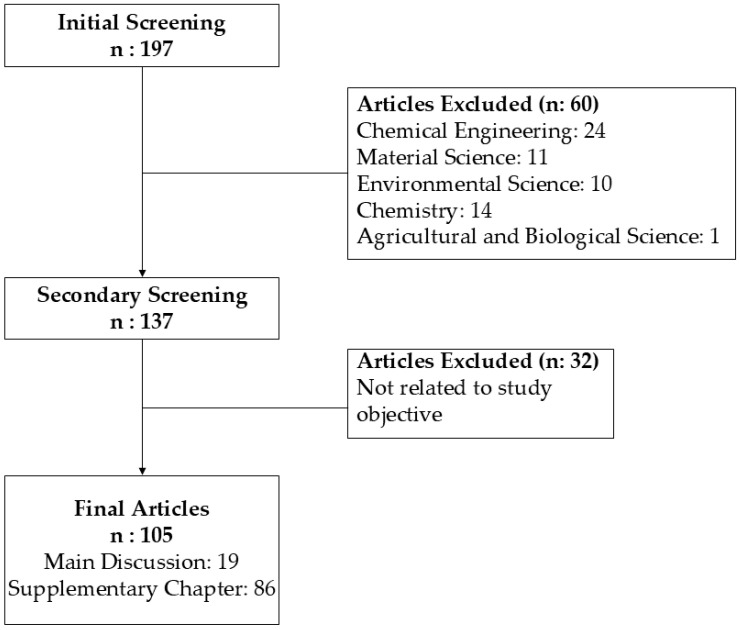
Flowchart of methodology.

This review applied a comprehensive and systematic approach to collect and analyze relevant data on improving the pharmacological efficacy of phytoconstituent in plant extracts by MSNs, as shown in Figure 1. A thorough search was conducted through the Scopus, Clarivate Analytics, and PubMed databases with keywords such as “mesoporous silica nanoparticles”, “ phytoconstituent”, and “drug delivery”, focusing on studies published from 1995 to 2024.

Stringent inclusion criteria were implemented, focusing on peer-reviewed studies that reported substantial enhancements in bioavailability, characterization, evaluation, and the therapeutic efficacy of plant extracts using MSNs, while excluding non-experimental, theoretical, or extraneous studies. The review used a systematic strategy, with each work carefully evaluated. This method ensured the inclusion of the most significant, reliable, and current result, establishing a solid basis for assessing the role of MSNs in enhancing the pharmacological efficacy of plant-derived drugs.

## 3. Medicinal Plant Extracts

Plants have been used as valuable sources of medicinal compounds for thousands of years, and many modern treatments have origins in natural sources. The increasing studies on worldwide biodiversity, a significant portion of which remains uncharted, emphasize the crucial significance of natural resources in satisfying medical requirements. Plant-derived chemicals have a molecular composition that leads to potential use for identifying new therapeutic targets. Pharmaceutical studies have an immense quantity of chemicals to explore, with the potential to uncover compounds with therapeutic properties [35]. However, the use of bioactive compounds in practical applications is limited due to issues such as inadequate water solubility, processing instability, and low bioavailability [36].

In general, plants offer a primary and plentiful source of natural therapeutic compounds used in traditional medicine. According to Özaslan and Oguzkan (2018), approximately 72,000 out of the total 422,000 species of flowering plants contain therapeutic properties [37]. Several studies have shown that phytochemicals derived from plants are reliable and have minimal adverse effects. Various botanical species have shown significant pharmacological properties, including anticancer, antibacterial, antidiarrheal, antioxidant, analgesic, and wound-healing activities (Table 1) [38]. Although herbal drugs and extracts offer the potential for use in pharmacology, their effectiveness in living organisms is often limited due to factors such as low solubility, limited absorption, and instability [39].

Medicinal plants have attracted significant attention in recent times due to their few adverse effects and rich concentrations of bioactive compounds [56]. This phenomenon could be related to the specific targeting of chemical components in drugs, resulting in advantageous therapeutic outcomes while minimizing negative reactions. Furthermore, medicinal plants have two crucial challenges in terms of using bioactive compounds for disease treatment and prevention: limited bioavailability and a short half-life. Large oral doses or frequent administration of the medication are often necessary to reach therapeutic levels in the bloodstream because the medication is not easily absorbed by the body [57,58,59]. Moreover, their significant hydrophobic nature, volatility, and lack of stability lead to a quick decline in effectiveness. To solve these issues, medicinal plant extracts can be encapsulated in various substances such as chitosan [4], alginate [5], liposomes [6], mesoporous silica [7,8], and other materials [60,61,62]. This could potentially maintain the therapeutic effectiveness for a longer period.

## 4. Mesoporous Silica Nanoparticles (MSNs)

Several studies have recently taken significant interest in the use of MSNs, which offer a distinct and potential drug delivery technique due to their consistent and adjustable pore size, capacity for independent surface modification, presence of both internal and external pores, and controllable pore opening mechanism. Specifically, the mechanism of controllable pore openings can be reached by different stimuli–response factors, such as pH, temperature, light, or enzymatic activity [63,64]. Previous studies have effectively used these carriers to encapsulate and transport a diverse range of substances, including proteins [63,64], DNA [65,66], RNA [13,67], and medicines. There is an extensive body of literature on this topic, and further studies are being conducted to discover novel applications of MSNs in the field of medicine administration. Several reviews have emphasized the roles of MSNs in enhancing drug solubility, facilitating controlled and prolonged drug release, as well as extensive uses in the field of biomedicine [68,69,70].

In general, there are organic and inorganic nanoparticles. Organic nanoparticles are carbon-based such as polymers, liposomes, dendrimers, micelles, nanogels, and layered biopolymers. Their characteristics include being naturally decomposed in the body, non-toxic, and friendly to biological systems. However, organic nanoparticles are very sensitive to light and heat, making their stability lower than inorganic nanoparticles which do not contain carbon, including silica-based nanoparticles, silicon, metal-organic frameworks (MOFs), and quantum dots [71]. The stability of inorganic nanoparticles is more stable chemically and physically. These nanoparticles are also suitable for medical applications due to their properties of being compatible with biological systems and the ability to load large amounts of drugs, as well as being resistant to extreme environmental conditions [72].

Among the widely used inorganic nanoparticles that are very effective as drug carriers are MSNs. The various types, including MCM-41, MCM-48, and SBA-15 with functionalization, are being considered highly promising options for drug delivery systems [73,74]. By using these carriers, the release or degradation of drugs in the pores and the deactivation effect of drugs before reaching the target can be prevented, allowing for controlled delivery [75].

MSNs have been widely used in various fields as sensors, catalysis, and biomedicine. For example, MCM-41 is often used to achieve high structural regularity and precise control [76]. MSNs are characterized by an irregular pore structure and particle sizes that vary depending on the synthesis method. Among several types, MCM-41 has a higher structural regularity and a more specific pore structure. It has a regular hexagonally ordered pore structure, narrow pore size distribution high surface area, and a highly organized cylindrical pore arrangement [77]. Moreover, MCM-41 is easily customized through surface functionalization and has been widely studied in biomedicine as a biosensor [78,79,80].

### 4.1. Mechanism of MSN Formation

To effectively combine MSNs with drug administration, it is essential to have a comprehensive understanding of their formation mechanism. Recent studies, particularly on materials produced from diluted surfactant solutions, showed that the silica network forms within non-ionic surfactants in liquid-crystalline phases. However, no mesostructured materials were found in these conditions. Published studies show that in the initial phases of development, hydrolyzed silica either attaches to the micelles or, in the case of SBA-15, interacts with the surfactant to form a structure resembling a core and shell [81].

The generation process of MSNs has been studied using time-resolved small-angle neutron scattering (SANS), a technology that allows for real-time detection of alterations. During the initial hydrolysis phase of tetramethyl orthosilicate (TMOS), silicate ions attach to the surfactant micelles within around 40 s. The first hydrolysis and condensation of silica precursor reduce the charge surrounding the surfactant, thereby decreasing intermicellar repulsion and facilitating the aggregation of small silica particles [82]. Transmission electron microscopy (TEM) verifies that the reaction mixture forms a suitably hexagonal mesoporous silica structure within approximately 400 s. These results are consistent with the previously hypothesized ‘current bun hypothesis’ for the formation of MSNs [83].

### 4.2. MSNs Can Improve Dissolution Rate

MSN pores have been used for the encapsulation of several pharmaceuticals and bioactive substances that have limited solubility in water. Previous studies have extensively investigated the release patterns of these compounds into the environment. The majority of the compounds belong to Biopharmaceutics Classification System (BCS) Class II, while there are also recorded examples from Classes I, III, and IV. Furthermore, previous studies have extensively examined the carrier materials of MCM-41 and SBA-15, known for well-organized, one-directional, and homogenous mesopores. Other structures of mesoporous silicas, such as SBA-16, MSF, MSU, COK-12, KIT-6, and FDU-12, have been identified and evaluated for comparison. These variables include the loading method, pore size, mesoporous structure, surface area, and surface functionalization that influence the drug-release profile from the matrix.

### 4.3. Effect of Pore Size

MSNs have been used as carriers for several pharmaceuticals and bioactive compounds that have low solubility in water. Extensive research studies have been conducted to investigate the release properties of these compounds in laboratories. Although there are additional examples from Classes I, III, and IV, the majority of the compounds are categorized as BCS Class II. The carrier materials that have been extensively studied include SBA-15 and MCM-41. These materials are known the well-structured, uniform, and one-directional mesopores. Additional structured mesoporous silicas that have been proposed and evaluated for comparison include SBA-16, MSF, MSU, COK-12, KIT-6, and FDU-12. A comprehensive study was performed on the variables that influence the release of the drugs from the MSN matrix, including the loading procedure, pore dimensions, mesoporous arrangement, surface area, and surface functionalization.

The drug release from MSNs is strongly influenced by the pore size (d*). Cheng et al. investigated the effect of pore size on drug release [84]. The release profiles showed that increasing the pore size from 4.5 to 6.4 nm greatly enhanced the release of the drug. These results show that there is a critical pore width that influences the steric barrier to drug diffusion in the pores. A previous study also reported the effect of three different types of silica material, which have pore widths varying between 20 nm and 40 nm on the physical state of active pharmaceutical ingredient (API). The analysis showed that although the larger pores made it easier for the medication to spread, the nanocrystalline drug contained within pores larger than 20 nm showed a reduced rate of dissolution. Compared to SBA-15 and MCM-41, the drug showed a slower disintegration rate in SBA-15-P due to the presence of larger ibuprofen particles.

### 4.4. Effect of Mesoporous Structure

Tortuosity (τ) refers to the connectivity and structure of pores, determined by the Higuchi equation as a critical factor that governs the diffusion of the drug molecules from a porous matrix. Xu et al. [76] conducted a comprehensive evaluation of this parameter by investigating the influence of the ibuprofen dissolution rate on different pore conformations (unidirectional, 2D, and 3D) and pore widths [85]. The study evaluated MCM-41 and SBA-15 as types of mesoporous silicas that are uniform and have pores in a single direction. It also investigated thermally carbonized porous silicon (TCPSi) as a two-dimensional mesoporous material with a broad range of pore diameters, ranging from 2 to 30 nm. TUD-1 (Technische Universiteit Delft) discovered a three-dimensional interconnected mesopore network (2.5–20 nm) with a sponge-like porous structure.

The release characteristics of the drug from the materials were evaluated at different pH levels to simulate the conditions of the small intestine. Every instance of using mesoporous materials led to a significant enhancement in the release of the drug compared to the drug in bulk, resulting in a consistently accelerated release. After 45 min, 25% of the total amount of API was dissolved in a phosphate buffer medium with a pH of 5.5. However, the presence of mesoporous carriers yielded a 2.5-fold to 3.5-fold increase in the release of the drug. TUD-1 showed the most rapid release due to the 3D mesopore network’s convenient accessibility. SBA-15, with characteristically larger pores, and MCM-41, distinguished by narrower channels, were introduced. Both effectively limit the diffusion of the drug molecules into the solvent. Among these materials, SBA-15 had the highest drug-loading capacity and fastest drug-release qualities, making it the most potential matrix for increasing dissolution. This property allows for easier formulation of large amounts of the drugs with increased dissolution rates.

### 4.5. Effect of Surface Area

Azevedo et al. [86] synthesized MCM-41 materials with varying porosities and surface areas and then examined the release profile of the drug from the matrices [29]. The results showed that once a certain degree was reached, there was no longer a significant correlation between increased surface area and enhanced dissolution. The concept of effective surface area is the primary determinant of the dissolution rate. The drug molecules were disseminated on silica carriers’ extensive surface area at the molecular, supramolecular, or particulate levels. However, increasing the surface area of the carrier did not lead to further subdivision of the drug due to increased cohesive forces and surface tension between the drug particles. The available surface area for in vitro dissolution was consequently reduced, leading to the lack of extra improvement in dissolving despite an increase in total surface area.

### 4.6. Effect of Surface Functionalization

Surface functionalization is a frequently used method to enhance the controlled release properties of mesoporous silica materials (MSMs). This occurs by facilitating specific interactions between functional groups on the carrier surface and the drug molecules enclosed within. Studies on the use of functionalized, ordered mesoporous materials to enhance medicine solubility rates are limited. The objective of surface functionalization is generally to achieve a reduced release rate [86,87]. For instance, the introduction of functional groups such as (basic) NH2 onto the surface of mesoporous silica might lead to specific interactions between host and guest molecules, particularly those containing acidic groups. However, COOH-functionalization can be used for mostly basic medicinal molecules. The acidity of silica is due to the inherent existence of silanols on its surface [81].

Strong contacts are preferred to stabilize the molecule in the amorphous state during the drug-loading phase. On the other hand, strong repulsion is preferred during the release phase to facilitate rapid drug release from the carrier matrix. The loading phase, which relies on intramolecular interactions such as hydrogen bonding, and the release phase, mostly determined by electrostatic interactions, can both be conducted in aqueous conditions to meet these contrasting needs [82]. When evaluating drug-release behavior, it is important to consider the silica surface, which may include residual silanols and be modified with acidic or basic groups. This is crucial because the majority of medications are either weak acids or bases.

Surface groups that are not ionizable can enhance the hydrophobicity of silica surfaces. This, in turn, will slow down the process of the aqueous solvent wetting the surface and increase the hydrolytic stability of the silica matrix, leading to a delay in the release of the drug. Insufficient wetting potentially hinders the release of hydrophobic guest molecules into water [83], specifically when there is an overwhelming amount of these molecules present. An exhaustive evaluation of these variables is essential to optimize the drug release profiles from functionalized MSNs.

### 4.7. MSNs Can Improve Drug Bioavailability

Mellaerts [88] conducted the first in vivo evaluation of MSMs with an ordered pore diameter of around 7.3 nm in rabbits and dogs to evaluate the bioavailability of itraconazole. The reference formulations used for comparison were crystalline itraconazole and the commercially marketed drug Sporanox^®^. The solubility rate of pure itraconazole was comparatively slower than the release in an aqueous media in vitro. In addition, a supersaturated solution was created in a situation when the solute concentration exceeded the equilibrium solubility. There was a significant enhancement in the bioavailability of itraconazole in both dogs and rabbits. When tested in rabbits, the crystalline form of itraconazole showed an area under the curve (AUC0-24) of 521 ± 159 nM·h and a Tmax of 9.8 ± 1.8 h during 24 h. In contrast, the itraconazole loaded in silica showed an improved AUC of 1069 ± 278 nM·h and a Tmax of 4.2 ± 1.8 h. Significant absorption of Sporanox^®^ occurred after it was administered.

Wang et al. [47] assessed the bioavailability of carbamazepine by using pellets made with SBA-15 and extruded/spheronized with appropriate excipients. The pharmacokinetic properties of carbamazepine-SBA-15 pellets were determined and compared to those of commercially available carbamazepine tablets following oral ingestion. The primary pharmacokinetic parameters, namely AUC, Cmax, and Tmax, of the carbamazepine-SBA-15 pellets, were found to be 113,709 ± 17,150 ng·h/mL, 803.7 ± 296.78 ng/mL, and 65.00 ± 12.25 min, respectively. The metrics for the commercial pills were 72,580 ± 25,283 ng·h/mL, 528.83 ± 106.85 ng/mL, and 65.00 ± 15.49 min, in the same sequence. The disparity in Cmax did not exhibit significance (*p* > 0.05) but the discrepancy in AUC was deemed significant based on the Student’s *t*-test.

A study conducted by Iranshahy [33] showed that curcumin loaded in MSNs has higher cellular uptake, subsequently enhancing antioxidant activity. Cao et al. [89] assessed the in vitro and in vivo correlation of Silybin meglumine and the results show that MSNs could achieve an eminent correlation between dissolution (in vivo) and absorption (in vivo). However, the analysis of cellular uptake could not be conducted since the mechanism of plant extracts is not targeted.

As for bioavailability, nanoparticles primarily enter cells through energy-dependent simple diffusion or translocation. Energy-dependent endocytosis, which enables the uptake of nanoparticles and submicron particles from an external environment to the cell plasma membrane, is the most prevalent process of internalization. The mechanisms can be classified in general into phagocytosis, pinocytosis, micropinocytosis, receptor-mediated endocytosis, clathrin-mediated endocytosis, caveolin-mediated endocytosis, flotillin, CDC42 (CLIC/GEEC)-dependent endocytosis, Arf-6, Rho-A, or IL2Rb-dependent pathways. After two hours of treatment, almost 80% of MSNs are trapped in the RES of the liver, spleen, and lung, according to quantitative measurements of biodistribution and clearance [90].

## 5. Development of MSNs of Medicinal Plant Extracts

Previous studies have reported the loading of medicinal plant extracts into MSNs, as shown in Table 2.

### 5.1. Preparation of MSNs for Medicinal Plant Extracts

An effective approach to enhancing drug release kinetics entails incorporating medicines into MSNs by solvent-based techniques including solvent evaporation, initial moisture impermeability, and adsorption. In this context, adsorption refers to the transfer of medications from an organic solvent to the porous structure of MSNs. This process entails immersing silica in the solution, leading to the adhesion of the drug molecules to the inner surfaces of the pores. The drug-loaded MSNs are subsequently isolated from the solution using filtration or centrifugation, and any residual solvent is eliminated. The incipient wetness impregnation approach enables precise evaluation of the drug loading by gradually adding a concentrated drug solution to silica and leveraging the high pore volume. Meanwhile, the solvent evaporation method combines rapid removal of the solvent with adsorption. To accomplish this, silica is dispersed in a volatile organic solvent that incorporates the drug, such as dichloromethane or ethanol, and subsequently subjected to rapid drying using a rotary evaporator [7].

### 5.2. Characterization

#### 5.2.1. Thermal Analysis

Thermogravimetric-differential thermal analysis (TG-DTA) is an analytical technique used to evaluate the loss of weight caused by drug degradation and the release of volatile components. This is achieved by subjecting the sample to controlled temperature increases. The purpose of this analysis is to calculate the percentage of total drug content. TG-DTA combines thermogravimetric analysis (TGA) and differential thermal analysis (DTA). TGA is often conducted using various heating rates to measure the changes in the mass of materials in relation to temperature or time. DTA records both exothermic and endothermic processes by quantifying the temperature difference between a sample and a reference during thermal activities. TG-DTA is capable of detecting temperatures associated with endothermic or exothermic reactions, including melting and phase transitions, as well as weight changes caused by dehydration or decomposition. Ciobanu et al. detected three phases of heat degradation in AOE polysaccharide extracts at natural pH (NN) and acidic pH (NA) using TG analysis [34]. The mineral residues were found to be 42% and 48% in the corresponding stages. The DTG curve for the NN sample exhibited two minor peaks and one major peak, showing a significant loss of mass. The TG curve showed two distinct stages of weight reduction after encapsulating AOE in MSNs (AO/MSNs), resulting in a mineral residue ranging from 21% to 41% depending on the concentration of extract. The release of hydrogen-bonded water from the polysaccharide and silica surface was shown by the initial decrease in weight below 150 °C. Gradual weight reduction started at 200 °C for NN and NA extracts, while for MSN-supported samples, it initiated at around 250 °C. The free extracts (NN and NA) were subjected to two stages of polysaccharide degradation, resulting from the decomposition of more resilient constituents. The second stage occurred at a temperature of 400 °C. Encapsulating the substance within MSNs led to an increase in decomposition temperatures, showing a correlation with silica support. The weight drop was significantly greater when the polysaccharide extract was prepared in an acidic environment, leading to a faster breakdown, compared to extraction in a neutral environment. The DTA curves of all samples initially showed a little endothermic region due to the release of adsorbed water then there was a transition towards an exothermic reaction with a distinct peak at around 300 °C. This peak showed the decomposition of polysaccharides into volatile gases in the presence of oxygen.

Azevedo et al. reported the thermal breakdown of MSNs (RP/MSN) with red propolis extract. The study confirmed the occurrence of an endothermic reaction, as evidenced by a single mass loss event observed between 80 and 100 °C. The remaining amount of water adsorbed in the nanopores was determined to be approximately 32.64%. The thermogravimetric curve of the red propolis extract showed three distinct points of mass decay namely dehydration (between 100 and 120 °C) and melting (between 120 and 135 °C) of low molecular weight compounds including flavonoids, isoflavonoids, as well as other phenolic compounds, and decarboxylation (between 220 and 450 °C) of the remaining organic matter. The RP/MSN TG curve showed two instances of degradation corresponding to the disintegration patterns observed in the preceding thermogram, following the initial decay event at 80 °C, which led to approximately 13.56% water loss. There was a mass loss of around 14.56% up to a temperature of 550 °C. The encapsulation of propolis led to an increase in the degradation temperature, suggesting enhanced physicochemical stability of the bioactive component [86].

**Figure 2:**
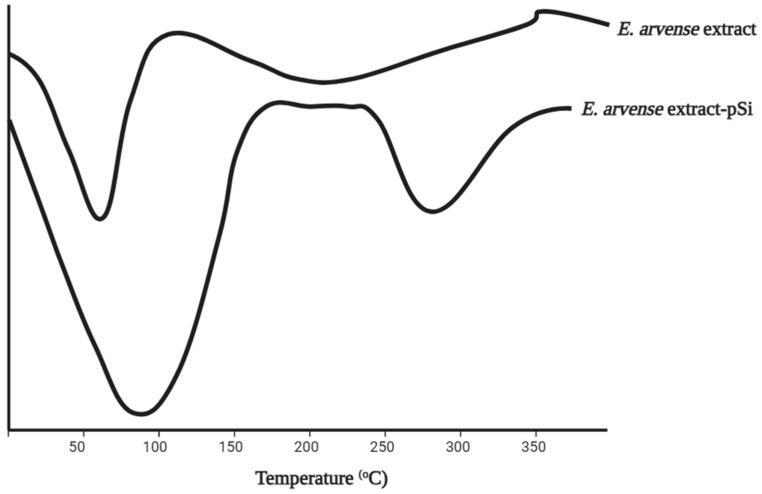
Overlay of DSC thermograms of E. arvense extract and this extract loaded into pSi. Adapted from data presented originally in Ref. [6].

Differential scanning calorimetry (DSC) is the most effective method for comprehending the physical attributes of bioactive compounds derived from MSNs. The thermal stability of these compounds was evaluated using a DSC fitted with an optical microscope. The specific heat change determined at the inflection point showed a type II glass transition characterized by examination of the AOE extract as a transition from an amorphous to a supercooled liquid state. However, the encapsulated extract did not experience any changes within the temperature range tested. Kalluri et al. (2019) performed DSC measurements on an EAE extract that was incorporated into MSNs [6]. The EA extract included two primary phenolic compounds, namely quercetin and kaempferol. DSC investigation showed the presence of two distinct endothermic processes occurring within temperature ranges of 50–150 °C and 200–350 °C as shown in Figure 2. The first phenomenon is associated with the melting of two primary phenolic compounds, quercetin and kaempferol, which have melting points of 276 and 316°C, respectively. Meanwhile, the second phenomenon is attributed to the evaporation of water, co-solvents, or residual extract components with low melting points. The melting peak of the EA extract, after being loaded into porous silicon (pSi), changed to a lower temperature range on the DSC trace. This shows that the size of the crystallite was reduced due to being confined within the pores.

#### 5.2.2. X-Ray Diffraction (XRD) Measurement

**Figure 3:**
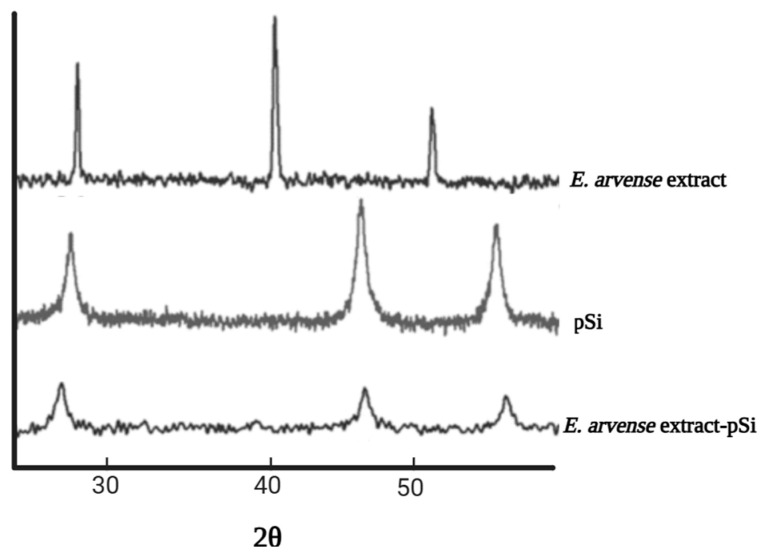
XRD analysis of E. arvense dry extract, before and after loading into pSi microparticles. Adapted from data presented originally in Ref. [6].

XRD analysis was conducted to assess the structural characteristics of MSNs before and during incorporation with plant extracts. The presence of p6 mm ordered hexagonal symmetry in MSNs was confirmed by observing three different peaks within the 0.8 < 2θ < 2 range. These peaks coincided with the (100), (110), and (200) reflections in the small-angle XRD patterns. The presence of plant extracts led to a reduction in the strength of these peaks, with a more pronounced effect observed in samples containing AOE (AO/MSN). The XRD diffractograms of dried EAE extract and EAE extract-loaded porous silicon (pSi) microparticles were also analyzed, as shown in Figure 3 [6]. The diffraction pattern of the EA extract had prominent peaks at 26°, 41°, 51°, and 67°, suggesting the existence of highly crystalline components in the mixture. However, if the concentration of the loaded extract falls within the detection limit of XRD, the absence of these peaks following the loading into pSi microparticles shows that the individual components of the extract may have experienced a transformation into nanostructures within the porous silicon matrix.

#### 5.2.3. Nitrogen Adsorption–Desorption Isotherms

The Brunauer–Emmett–Teller (BET) method is used to calculate the volume of gas adsorbed at standard temperature and pressure (Vm), the total pore volume (Vtot), and the average pore diameters (dp). Conversely, the Barrett–Joyner–Halenda (BJH) method is used to define the mean pore area (ap), pore volume (Vp), peak radius (rp), and pore size distribution curves. This study assessed the pore size properties of MSNs modified with amino groups (MSN-NH2) and loaded with GSE (GSE/MSN-NH2) using the BJH and BET models, respectively. Based on the result, GSE/MSN-NH2 had lower values for Vtot, Vm, dp, Vp, and ap, and slightly lower values for rp compared to MSN-NH2. This was probably because GSE was already loaded within the MSN-NH2 matrix

After loading the plant extract, the nitrogen adsorption/desorption experiments showed a significant reduction in both pore volume and surface area. The samples labeled as SNNIII and SNAIII exhibited a showed a significant reduction in pore size together with an enlargement of the range of pore sizes, leading to a modification of the adsorption-desorption isotherms. These results suggest that the constituents of polysaccharides infiltrate the pores, causing blockages in the micropores and depositing molecules in the mesopores. The diverse chemical makeup of these extracts does not exclude the possibility of larger polysaccharide components that extend beyond the limits of the pores. Elevated loading enhances the interactions between polysaccharides, leading to the accumulation of adsorbed layers outside the SBA-15 pores. This accumulation obstructs access, resulting in a considerably reduced pore volume and surface area. However, the impact of SBI samples on MSNs’ textural properties was less pronounced compared to SNNIII and SNAIII, despite SBI using a more concentrated loading solution. This discovery underscores the efficient penetration of polyphenols into the pores and the minimal effect on adsorption on the pore walls. The hydroxyl (OH) groups present on the silica surface and the functional groups present in organic molecules facilitate hydrogen bonding and van der Waals intermolecular interactions. These interactions play a crucial role in the adsorption of organic compounds from plant extracts.

Buda et al. reported that phytochemicals from black chokeberry can adsorb into the mesopores (AM/MSN) of silica-based supports, leading to a considerable reduction in porosity [42]. The specific surface area of MSN-pristine and MSN-Zn, as calculated by the Brunauer–-Emmett–Teller method (BET), decreased from 781 and 620 m^2^/g to 89 and 17 m^2^/g, respectively. Similarly, the total pore volume (Vpore) decreased from 0.78 cm^3^/g to 0.12 cm^3^/g and from 0.80 cm^3^/g to 0.11 cm^3^/g, respectively. There was also a decrease in the average pore diameter (dBJH), from 2.81 nm in MSNs to 2.52 nm in AM/MSNs. This shows that the phytochemicals from Aronia have been absorbed into the pores of silica-based substrates.

#### 5.2.4. Scanning Electron Microscopy (SEM)

Nanoparticles can be characterized using SEM. The variations in particle size values obtained through SEM can be attributed to discrepancies in sample preparation, namely the dry state of particles during the SEM analysis. SEM images of MSNs and led with red propolis extract (RP/MSN) were acquired at magnifications of 15,000× and 18,000×, respectively, using a scale bar of 1 μm. The photomicrographs showed particles spherical in shape and have diameters varying between 100 and 300 nm. However, due to challenges in controlling the rate of nucleation and development during the initial formation process, particle aggregation led to an irregular distribution of final sizes. Si-Han Wu and Lin showed that the morphological attributes and particle dimensions were contingent upon the kinetics of sol-gel chemistry, as well as the reaction temperature, pH level, and concentrations of water, surfactant, and silica precursor. Despite these limitations, the propolis extract was effectively absorbed. The uneven dispersion of the ultimate particle size may have been attributed to the calcination method used for the elimination of cetyltrimethylammonium bromide (CTAB).

Surface morphology analysis was conducted using field emission scanning electron microscopy (FE-SEM). The images showed a higher concentration of nanoparticles within the polymeric matrix, particularly mesoporous ones. Silica-free samples have a consistently even surface, in contrast to the samples containing silica nanoparticles. Previous morphological investigations have shown that the introduction of silica nanoparticles led to the development of an uneven and rough surface. The dispersion of nanoparticles within the biocomposite structure plays a crucial role in facilitating the process of wound healing. The acquired images confirm the formation of a three-dimensional nanoparticle architecture, which enhances the contact area and efficacy of wound dressings [87,91]. The results also show that contact with the aqueous extract can dry up the surrounding area, leading to the fragmentation of the samples. Conversely, samples without medication but with mesoporous silica did not experience any cracking, showing the strengthening role of the polymeric matrix. Sample S5 contained MSNs made up of carboxymethyl chitosan-gelatin at a concentration of 2.5%. These nanoparticles possess a well-defined grain boundary and maintain individual, non-agglomerated structures, suitable for use in medical applications [92]. The diagram shows the geometric arrangements of MSNs embedded in the polymer substrate. This shows that the process of pore filling decreases the scattering of nanoparticles in samples loaded with extract.

#### 5.2.5. Fourier Transform Infrared Spectroscopy (FTIR)

FTIR is an essential analytical method for identifying functional groups in substances by identifying certain peak distributions in the infrared spectrum [5,7]. This method is commonly used to validate the interactions between extracts and silica surface, as well as to investigate the chemical states of extracts within MSNs [88].

In a study conducted by Shahriarinoir et al., the characterization of GSE loaded into MSNs (GSE/MSNs) showed specific spectral features at 1390 cm^−1^, 1413 cm^−1^, 1550 cm^−1^, 1641 cm^−1^, 2893 cm^−1^, 3072 cm^−1^, and 3365 cm^−1^. These features show that the GSE was effectively loaded onto MSN-NH₂. The GSE/MSN spectra showed a minor displacement of the peak (~1 cm^−1^), providing additional evidence for the loading. In addition, the MSNs showed Si-O-Si bending, symmetric, and asymmetric stretching vibrations at frequencies of 484 cm^−1^, 796 cm^−1^, and 1081 cm^−1^, respectively [41].

Azevedo et al. conducted a study on red propolis extract loaded into MSNs (RP/MSNs) and found that the hydroxyl groups of silica as well as the phenolic compounds in propolis showed hydrogen bonding interactions. The FTIR signal at 3450 cm^−1^ showed a substantial drop, suggesting the occurrence of interaction. The structural integrity of MSNs containing red propolis was confirmed by the existence of characteristic peaks at 1240 cm^−1^, 1076 cm^−1^, 953 cm^−1^, 812 cm^−1^, and 450 cm^−1^ [86].

**Figure 4:**
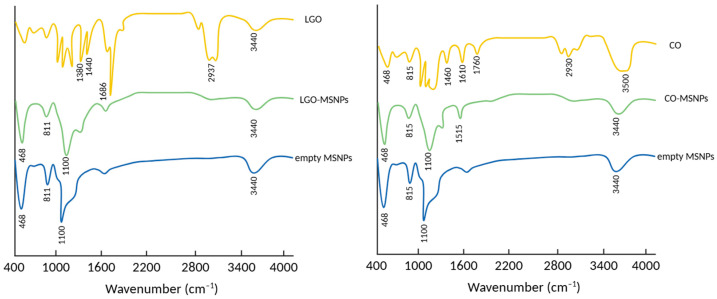
The characterization of grape seed extract loaded into MSNs (GSE/MSN). Adapted from data presented originally in Ref. [52].

Sattary et al. further investigated the use of MSNs for encapsulating clove oil and lemongrass oil. Citral, known as lemongrass oil, and eugenol, also called clove oil, exhibited clear absorption peaks at 1686 cm^−1^ and 1430 cm^−1^, respectively. The bending and stretching vibrations of the Si-O-Si groups were observed at frequencies of 468 cm^−1^, 811 cm^−1^, and 1100 cm^−1^ as shown in Figure 4 [52]. The presence of essential oils from lemongrass (LG) and clove (CV) within MSNs was shown without any harm to the MSN structure. This was confirmed by the detection of characteristic peaks in the spectra of (LG/MSNPs) or (CV/MSNPs).

#### 5.2.6. Solubility and Dissolution Rate Study

The solubility study found that MSNs significantly enhanced the rate at which natural components dissolve compared to extracts of those chemicals alone. The study investigated the solubility of various natural compounds, including grape pomace and EAE (E. arvense). 

Brezoiu et al. found that the release of phytochemicals from grape pomace-MSNs was significantly higher compared to the polyphenolic extract alone. The release structure of polyphenols from both unmodified and modified MSN carriers showed a partial restoration of polyphenols, with MSN-COOH indicating the highest restoration rate at 73.5 ± 1.3%, while pure MSNs showed the lowest restoration rate at 57.8 ± 0.6%. This shows that the surface properties of silica significantly influence the efficacy of releasing polyphenols and their biological effects [47].

Kalluri et al. discovered that the dissolution of E. arvense–MSNs in water enhanced the release of phytochemical extracts compared to the pure extract. The quantity of soluble extract released from porous silicon (pSi) within a mere 3.5 min was significantly higher compared to the amount obtained from the bulk material. A significant disparity (>10%) in the quantity of soluble extract generated from pSi compared to extract alone was observed at later time intervals (3.5 to 143.5 min) [6]. These results show that the solubility of the extract was improved due to the confinement of the individual components within the nanostructured pores, as confirmed by DSC and XRD investigations. The soluble extract from pSi released a cumulative amount that was twice greater at the end of 143.5 min compared to when the extract was used alone. The results show the potential of MSNs to enhance the bioavailability and solubility of natural compounds.

#### 5.2.7. In Vitro and In Vivo Evaluation

In vitro studies have shown that the antioxidant properties of extracts are greatly enhanced by the inclusion of MSNs. The improved performance can be attributed to the regulated release mechanism of MSNs, ensuring a consistent and prolonged release of polyphenols at optimal concentrations, and enhancing their antioxidant efficacy. Moreover, the addition of MSNs significantly enhanced the antibacterial effectiveness of extracts. Indicators of this included a lower MIC value and a larger zone of inhibition. The controlled release of active compounds from MSNs improves the effectiveness of antimicrobial drugs by maintaining high concentrations near the target bacteria for long periods. Previous studies have shown that the inclusion of MSNs in extracts enhances the ability to cause cell death and inhibit the movement of melanoma cells. The migration of melanoma cells is significantly hindered by the controlled and gradual release of active substances from microstructured supernatants, underscoring the potential of MSNs to enhance the effectiveness of bioactive chemicals in therapy.

Safat et al. conducted a study to determine the hepatoprotective and cholesterol-lowering effects of MSN-containing extract from Cynara scolymus. The results showed a significant reduction in triglyceride, total cholesterol, and very low-density lipoprotein (VLDL) levels compared the administration of C. scolymus extract alone. This improvement was attributed to the enhanced medicine delivery facilitated by MSNs [41]. In another study conducted by Haririan et al., administering Myrtus communis L. extracts to MSNs at a dosage of 5% resulted in significant wound-healing effects. The size of wounds and edema in mice was considerably reduced on days 7 and 12 of observation compared to mice treated with lesser dosages (1.25% and 2.5%) of M. communis extracts loaded on MSNs. The group that received 5% showed comparable effectiveness to the group given nitrofurazone treatment, indicating that MSNs loaded with medicinal plant extract possess enhanced therapeutic capacity [49]. The results emphasize the promising potential of using MSNs to efficiently convey medicinal plant extracts, enhancing the therapeutic effectiveness in many applications.

## 6. Discussion

The high hydrophobicity, volatility, and low stability of compounds lead to the loss of activity before completing specific action. Therefore, it is necessary to encapsulate the medicinal plant extract into different matrices, such as mesoporous silica [40]. Several characterizations, including FTIR, XRD, nitrogen adsorption–desorption isotherm, and TGA, showed the success of loading extracts into MSNs. The FTIR analysis showed that the main peaks of the extract were present in the loaded MSNs. This means that the extract was successfully loaded into mesoporous silica without damaging the structure [39]. In addition, the intensity of the peak in XRD decreased following the loading of plant extracts. A very low intensity was obtained for the highest loading, showing that MSNs can entrap extract in a more soluble form [41]. The analysis result obtained using DSC showed that an extract loaded with MSNs did not show any melting peaks [44]. On the other hand, the analysis using TGA showed that the mass of extract loaded into MSNs decreased within a certain temperature range. The loaded extract also showed a change in decomposition temperature toward higher values. The encapsulation of the extract into all channels of the MSNs was proved by a decrease in surface area (as determined by BET), average pore diameters (dp), total pore volume (Vtot), and volume of gas adsorbed at STP (Vm) [41]. However, given that the extract contains multiple compounds, these characterizations cannot investigate the molecular state of each compound. The characterization only shows whether the extract is already loaded in the MSNs or not. 

After confirming that the extract was already loaded into the MSNs through characterization, the dissolution study was used to analyze the release. The result showed that MSNs have great potential to significantly enhance the dissolution rates of various extracts compared to the pure forms. This improvement can be attributed to the distinct properties of mesoporous silica, including the high surface area and adjustable pore size. Extract dispersed monomolecularly within the MSNs. The dissolution medium entered immediately after the extract was dispersed in the MSNs. The monomolecularly dispersed extract rapidly dissolves and releases into the bulk dissolution medium. The rapid dissolution of extract led to the formation of a high concentration or supersaturation level in the dissolution medium. These properties help in the better dispersion and release of encapsulated phytochemicals. An extract can be entrapped into MSNs in a more soluble form presumably owing to the nanostructure of extract components. This shows that mesoporous morphologies of extract-loaded MSNs can load a complex multicomponent mixture in a more soluble form, thereby ideally enhancing the bioavailability [6]. Furthermore, the adsorption of the drugs onto the MSNs enhances wettability and increases the surface area available for dissolution, resulting in a faster rate of drug release [93]. In dissolution studies, the phenomenon of incomplete release or solvation of natural compounds from MSN-loaded herbal extracts or single-compound systems is a recognized issue in drug delivery studies. This behavior arises from the strong interactions between certain compounds and mesoporous silica matrix, which may lead to partial entrapment within the pore structures. These interactions can include hydrogen bonding, van der Waals forces, or even covalent bonding in cases where the functionalization of silica surface creates reactive groups presumably occurring with compounds that possess multiple hydroxyl or polar groups. These groups can establish a robust interaction with the silica surface, leading to slower or incomplete release profiles. However, studies consistently show that the extent of such entrapment is relatively minor and does not significantly affect the overall solubility or bioavailability enhancement achieved by the MSN system. This is because the enhanced surface area, improved wettability, and nanoscale dispersion provided by MSNs overwhelmingly compensate for the limitations. The solubility, dissolution, and release behavior remain significantly improved compared to the pure compound, ensuring a marked increase in bioavailability. Additionally, the controlled pore structure of MSNs plays a crucial role, as it prevents rapid aggregation of hydrophobic compounds and facilitates sustained release, which further supports their effectiveness as drug delivery systems. By addressing this challenge through careful optimization of MSN synthesis, pore size, and surface functionalization, future developments can continue to mitigate entrapment issues while leveraging the advantages for improved pharmacological performance.

**Figure 5:**
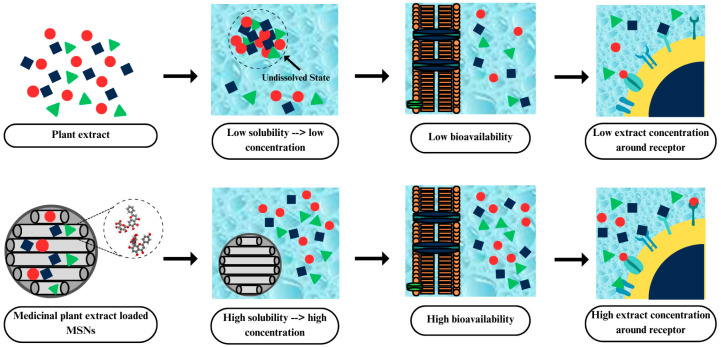
Mechanism of MSNs.

The pharmacological activity of medicinal plant extracts loaded with MSNs is more effective compared to medicinal plant extracts alone due to nanosized particles, as shown in Figure 5. The encapsulation of medicinal plant extracts into MSNs can increase the solubility and stability, facilitating gradual release into the environment and leading to the improvement of bioavailability. Passive diffusion generally achieves drug absorption, where drugs diffuse along the concentration differential from a higher to a lower concentration until equilibrium is reached. Incorporating extract into MSNs allows the soluble extract in the body’s watery parts, such as the interstitial space, to pass through water-filled pores in the endothelium of blood vessels. Therefore, the solubility of extract in the aqueous compartment of the body significantly affects the amount of extract absorption through passive diffusion. The monomolecularly dispersed extract improved solubility and created a supersaturated solution in the body’s aqueous compartment, which increased bioavailability. Improving the bioavailability of extracts could also enhance pharmacokinetics, efficacy, and safety. Therefore, the encapsulated state of the medicinal plant extract has greater pharmacological activity compared to the pure state, resulting in a lower dosage of medicinal plant extract needed [94,95,96].

## 7. Conclusions and Future Perspectives

### 7.1. Conclusions

In conclusion, the incorporation of plant extracts and MSNs greatly improves the effectiveness, stability, and ability of herbal formulations to be absorbed by the body, representing a groundbreaking development in the field of medicinal delivery systems. The successful integration and optimum interaction of bioactive compounds within a silica matrix are confirmed using thorough characterization methods, such as XRD, DSC, FTIR, TGA, SEM, and TEM. This validation ensures enhanced solubility and precise control over the release rate of the compounds. These improvements not only extend the duration of therapeutic effects but also optimize the biological activity and cellular absorption of plant extracts.

### 7.2. Future Perspectives

Despite ongoing obstacles such as the scaling of production and the analysis of complicated multi-compound interactions, future interdisciplinary studies offer the potential for creative solutions. New technologies, such as nanomedicine and artificial intelligence-based formulation design, are expected to enhance delivery methods, providing improved precision and efficacy. The ongoing exploration of MSNs presents a promising opportunity for the creation of innovative delivery systems that could transform plant-based medicine, resulting in more efficient, stable, and safer therapeutic alternatives, thereby initiating a new era of personalized and advanced healthcare.

Future development of MSNs for plant extracts should build on advancements made in single-compound MSN systems while addressing the unique challenges posed by the complex, multi-component nature of herbal extracts. Current efforts for single-compound MSNs have shown remarkable progress in responsive drug delivery systems, such as stimuli-responsive release triggered by pH, temperature, or enzymes, enabling precise therapeutic action at target sites. These responsive mechanisms offer immense potential for herbal extracts but remain largely unexplored due to the heterogeneity of these extracts, which complicates the design of uniform stimuli-sensitive systems. Similarly, active targeting approaches, which are well-established for single compounds through ligand-functionalized MSNs, must overcome challenges related to compound competition and varied interactions within multi-component extracts. Future studies should explore the application of dual-functional MSNs capable of simultaneous solubility enhancement, sustained release, and selective targeting for a broader range of phytoconstituents. Advanced surface engineering methods, such as dynamic functionalization or co-delivery systems, could help achieve this goal. Furthermore, cellular uptake studies and biodistribution assays should be prioritized to better understand the interactions between MSN-loaded herbal extracts and biological systems, ensuring efficient delivery and distribution to target sites. The integration of intelligent drug delivery strategies, such as external stimuli-responsive MSNs (magnetically or light-triggered systems), could further enhance the therapeutic precision of MSN-loaded extracts. By leveraging these innovations and adapting to the complexity of herbal systems, the next generation of MSNs could transform phytotherapy into a highly efficient and targeted modality, bridging traditional medicine and advanced nanotechnology to address unmet clinical needs.

## Figures and Tables

**Table 1:** Pharmacological activity of active substances in different extracts.

No.	Extract	Active Substance	Pharmacological Activity	Extract Preparation	Ref.
1.	*Melissa officinalis* extract (MOE)	Polyphenol	Antimicrobial, antibacterial, cytotoxic, antidepressant, neuroprotective, cardioprotective, anti-inflammatory	The aerial part of *Melissa officinalis* was harvested and subsequently dried at 40 °C until a humidity level of 8% was achieved. The dried *Melissa officinalis* was then ground and extracted using 50% ethanol in water. The resulting liquid extract was concentrated using a nitrogen stream.	[40]
2.	Grape seed extract (*Vitis vinifera*) (GSE)	Polyphenol	Antioxidant, cardioprotective, hepatoprotective, neuroprotective, antidiabetic, anticarcinogen, anti-aging, antibacterial, and anti-inflammatory	The GSE was prepared by Zardband Pharmaceuticals Company.	[41]
3.	Black chokeberry extract (*Aronia melanocarpa*) (AM)	Polyphenols and flavonoids	Antioxidant, anti-inflammatory, antimicrobial (antibacterial and antiviral), antidiabetic, antiproliferative, hypotensive, anti-atherosclerotic, antiplatelet, cardioprotective, gastroprotective, immunomodulatory, and antitumor	A finely ground powder of dried *Aronia* berries was macerated at room temperature, followed by reflux heating. The resulting extract was then dried under a vacuum.	[42]
4.	Artichoke extract (*Cynara scolymus*) (CS)	Chlorogenic acid, caffeic acid, luteolin, cynarin	Antioxidant	-	[43]
5.	Sumac extract (*Rhus coriaria*) (RC)	Tannin	Anti-atherosclerosis	The sumac fruit was harvested and dried at room temperature for 24 h, followed by washing with distilled water. The dried fruit was then ground and extracted three times using ethanol under dark conditions. The resulting extract was then filtered using a Buchner funnel then dried in an oven at 33 °C for 48 h.	[44]
6.	Savory essential oil (*Satureja hortensis*) (SH)	Carvacrol	Anticancer effects observed in various cancer cell lines, including melanoma, breast cancer, and chronic myeloid leukemia	The Savory essential oil was extracted using the steam distillation method.	[45]
7.	*Althaea officinalis* extract (AOE)	Phenolic compound	Anti-inflammatory for various mucosal inflammations	Powdered *Althaea* leaves were extracted using distilled water at room temperature for 60 min. The concentrated extract was obtained using a continuous agitation system.	[34]
8.	Wild bilberry extract *(Vaccinium myrtillus*) (VM)	Anthocyanins	Antioxidant, anticarcinogenic, cardioprotective, anti-inflammatory, hypoglycemic, antimicrobial, and vision-improving effects	Extracts of wild bilberry fruits were prepared in acidified ethanol at 80 °C using a conventional method.	[46]
9.	Grape pomace extract (MM)	Polyphenolic compound	Antioxidant, anticarcinogenic, anti-inflammatory, antidiabetic, antibacterial, and cardioprotective	The hydroalcoholic polyphenolic extracts were prepared from the Mamaia cultivar using both conventional extraction and microwave-assisted extraction methods.	[47]
10.	Common sage extract (*Salvia officinalis* L.) (SO) and wild thyme extract (*Thymus serpyllum* L.) (TS)	Polyphenols and flavonoids	Effectiveness against a variety of fungi species and bacterial strains by antibacterial and antifungal agents	The ethanolic and hydroalcoholic polyphenolic extracts from the dried leaves of *Salvia officinalis* were prepared by conventional and microwave-assisted methods.	[48]
		Essential oils, phenolic acids, flavonoids	Anti-inflammatory	The ethanolic and hydroalcoholic extracts of *Thymus serpyllum* were prepared by a conventional method.	
11.	*Myrtus communis* L. extract (MC)	Phenolic acids, flavonoids, tannins	Antioxidant, antihemorrhagic, analgesic, antimutagenic, anti-inflammatory, hepatoprotective, antihyperglycemic activities, and wound healing	The aqueous extract of *Myrtus communis* dried leaves was obtained by the maceration method.	[49]
12.	Red propolis extract (RP)	Flavonoids, benzophenones, terpenes	Antimicrobial, anti-inflammatory, antioxidant, and antiproliferative	^-^	[48]
13.	Eucalyptus essential oil (EUC)	Terpenoid	Antibacterial effects against *Escherichia coli*, *Bacillus subtilis*, *Staphylococcus aureus*, *Listeria innocua*, and *Pseudomonas aeruginosa*	The essential oil was purchased from a local supplier.	[50]
14.	Essential oil lemongrass (LG) and clove (CV)	Terpenoid	Antimicrobial and antifungal	The essential oil was purchased from Zardband Pharmaceuticals Company.	[51]
15.	Lavender oil (*Lavandula angustifolia*) (LA)	Terpenoid	Antimicrobial and antifungal	Lavender essential oil was purchased from HauYon Co., Ltd.	[52]
16.	*Salvia officinalis* extract (SO)	Ursolic, rosmarinic, caffeic, and oleanolic acids	Anti-inflammatory	The ethanolic and hydroalcoholic polyphenolic extracts from the dried leaves of *Salvia officinalis* were prepared by conventional, microwave, and ultrasound-assisted extraction methods.	[53]
17.	*Equisetum arvense* extract (EA)	Sterols, phenolic acids	Antimicrobial and antioxidant	The ethanolic extract of *Equisetum arvense* leaves was obtained using a Soxhlet extractor, followed by the concentration of extract through a rotary evaporator.	[54]
18.	*Salvia officinalis* extract	Polyphenols	Antibacterial and antiproliferative	The polyphenol extract of *Salvia officinalis* was obtained using an ultrasound-assisted extraction at 40 °C in absolute ethanol. Then, the resulting extract was evaporated using a rotary evaporator.	[55]

**Table 2:** Current development of MSNs for medicinal plant extracts.

No.	Extract	Type of MSN	Preparation Method	Dissolution Study	Pharmacological Activity		Ref.
					In Vitro	In Vivo	
1.	*Melissa officinalis* extract (MOE)	MCM-41 and MCM-48	Adsorption		MSNs loaded with MOE have a much lower minimum inhibitory concentration (MIC) compared to MOE alone, showing enhanced effectiveness.		[40]
2.	Grape Seed (*Vitis vinifera*) extract	SBA-15-NH2	Adsorption	Within the initial hour, the GSE that was injected into SBA-15-NH2 was released at a rate of around 7.2%. After a total of 82 h, the overall percentage of release increased to 97.5%.			[41]
3.	Black Chokeberry (Aronia melanocarpa) extract	MCM-41	Incipient wetness impregnation			The addition of black chokeberry extract to mesoporous silica-type matrices significantly enhanced its antibacterial activity compared to using extract alone.	[42]
4.	Artichoke (Cynara scolymus) extract	SBA-15-NH2 and SBA—cysteine	Adsorption			The group treated with extract-loaded MSNs exhibited a significant decrease in triglyceride (TG), total cholesterol (TC), and very-low-density lipoprotein (VLDL) levels compared to the group treated alone with extract.	[43]
5.	Sumac (*Rhus coriaria*) extract	MSN-NH2	Adsorption		MSN-NH2/RC’s drug release profile in acidic environments shows a quick release of the medication.		[44]
6.	Savory (*Satureja hortensis*) essential oil	KC	Solvent evaporation		Compared to the injection of Savory extract alone, nanoformulations considerably increased the anticancer activity against HL60 leukemia cells and HepG2 liver cells.		[45]
7.	*Althaea officinalis* extract (AOE)	SBA-15 MSN	Incipient wetness impregnation		At 100–1000 μg/mL, Althaea extract had higher cytotoxic effectiveness against the Hep-2 tumor cell line than Betonica extract, which also caused morphological alterations at 200–1000 μg/mL.		[34]
8.	Wild bilberries (*Vaccinium myrtillus*) extract	MCM-41, MCM-SH, MCM-COOH, MCM-CN	Impregnation		The radical scavenging activity was shown to be more stable in extract loaded into MSNs than in its free state.		[46]
9.	Grape pomace extract	MCM-41	Incipient wetness impregnation	MM@MCM-COOH material had the greatest amount of phytochemicals released when compared to polyphenolic extract alone, and this material also showed the lowest cytosolic production of ROS.	Compared to the polyphenolic extract alone, the polyphenols-loaded MSN, specifically the MM@MCM-COOH sample, showed greater in vitro antioxidant activity and increased stability over time.		[47]
10.	Common sage (*Salvia officinalis* L.) and wild thyme (*Thymus serpyllum* L.) extract	MCM-41	Incipient wetness impregnation		Polyphenolic extracts from *Thymus serpyllum* L. and *Salvia officinalis* L. show greater radical scavenger activity when loaded into MCM-41 type silica than when extracts alone.		[48]
11.	*Myrtus communis* L. extract	MSN	Adsorption			The wound size was considerably (*p* < 0.05) smaller in the biocomposite dressing S3 (5% MSN) and nitrofurazone treatment groups.	[49]
12.	*Artemisia argyi* extract	MSN	Adsorption			The loaded AE into GelMA/HAMA has anti-inflammatory and antibacterial activities for application in the faster recovery of chronic wounds.	[5]
13.	Red propolis extract	MCM-41	Adsorption		The antibacterial activity of the Pr/MCM-41 was greater when compared to the free extract.		[86]
14.	Eucalyptus essential oil	SiO_2_	Solvent evaporation		SiO_2_-EUC had the highest activity against C. albicans, with MIC of 0.18 µg/mL against this yeast.		[51]
15.	*Lemongrass* and clove essential oil	MSN	Adsorption		Controlled release of lemongrass and clove essential oil.		[52]
16.	Lavender (*Lavandula angustifolia*) essential oil	MSN	Adsorption		Delayed volatilization of lavender essential oil.		[53]
17.	*Salvia officinalis*	MCM-41	Incipient wetness impregnation		In comparison to the free extract, extract stored at 4 °C for 2 to 12 months showed increased radical scavenger activity when loaded into MSNs.		[54]
18.	*Equisetum Arvense* extract (EAE)	Porous silicon	Adsorption	More soluble form, thus optimally enhancing its bioavailability.			[6]
19.	*Salvia officinalis* extract	MSN composite, each functionalized with -NH_2_ and -COOH	Impregnation, solvent evaporation		Polyphenolic extracts loaded into MSNs showed effective radical scavenging activity and enhanced antibacterial activity against both types of Gram bacteria. The composite form made from collagen exhibited prominent stability against the development of fungus.		[54]

## Data Availability

There were no new data created.
